# Field switching of microfabricated metamagnetic FeRh MRI contrast agents

**DOI:** 10.1038/s41598-025-85384-6

**Published:** 2025-01-22

**Authors:** Stephen Dodd, Natalia Gudino, Oleksii Zadorozhnii, Michal Staňo, Jan Hajduček, Jon Ander Arregi, H. Douglas Morris, Vojtěch Uhlíř, Mladen Barbic, Alan P. Koretsky

**Affiliations:** 1 Laboratory of Functional and Molecular Imaging, National Institute of Neurological Disorders and Stroke, National Institutes of Health, Bethesda, MD 20892 USA; 2 Central European Institute of Technology, Brno University of Technology (CEITEC BUT), Brno, 612 00 Czechia; 3 Institute of Physical Engineering, Brno University of Technology, Brno, 616 69 Czechia; 4 Department of Radiology and Radiological Sciences, Uniformed Services University of the Health Sciences, Bethesda, MD 20814 USA; 5 Tech4Health Institute, NYU School of Medicine, New York, NY 10016 USA

**Keywords:** Metamaterials, Imaging techniques and agents, Biomedical engineering

## Abstract

In a step towards generating switchable MRI cellular labels, we demonstrate in-situ field switching of micron scale metamagnetic Iron-Rhodium (FeRh) thin film particles. A thin-film (200 nm) FeRh sample was fabricated and patterned into an array of progressively smaller squares with sizes ranging from 500 μm down to 1 μm. The large first order phase change from antiferromagnetic to ferromagnetic state was characterized using vibrating sample magnetometry, magnetic force microscopy, and MRI. Room temperature MRI experiments sensitive to the local magnetic field surrounding the particles demonstrated the low moment state (OFF MRI contrast) at 4.7T and high moment state (ON MRI contrast) at 11.7T for the array where sizes down to 2–3 μm were observed in MRI at 50 μm resolution. The expected temperature dependent MRI contrast change was seen at 4.7T, where 10 μm particles could be observed at 150 μm resolution in the ON state. A shielded MRI insert, used to temporarily increase or decrease the magnetic field up to 0.77T amplitude, was used to reversibly switch the particle array at constant temperature and blink the particles ON and OFF at 4.7T. This work demonstrates the MRI contrast switching potential for FeRh particles with biological cell dimensions, and the use of magnetic field pulses for reversible MRI label contrast control.

**Supplementary Information:**

The online version contains supplementary material available at 10.1038/s41598-025-85384-6.

## Introduction

**Fig. 1:**
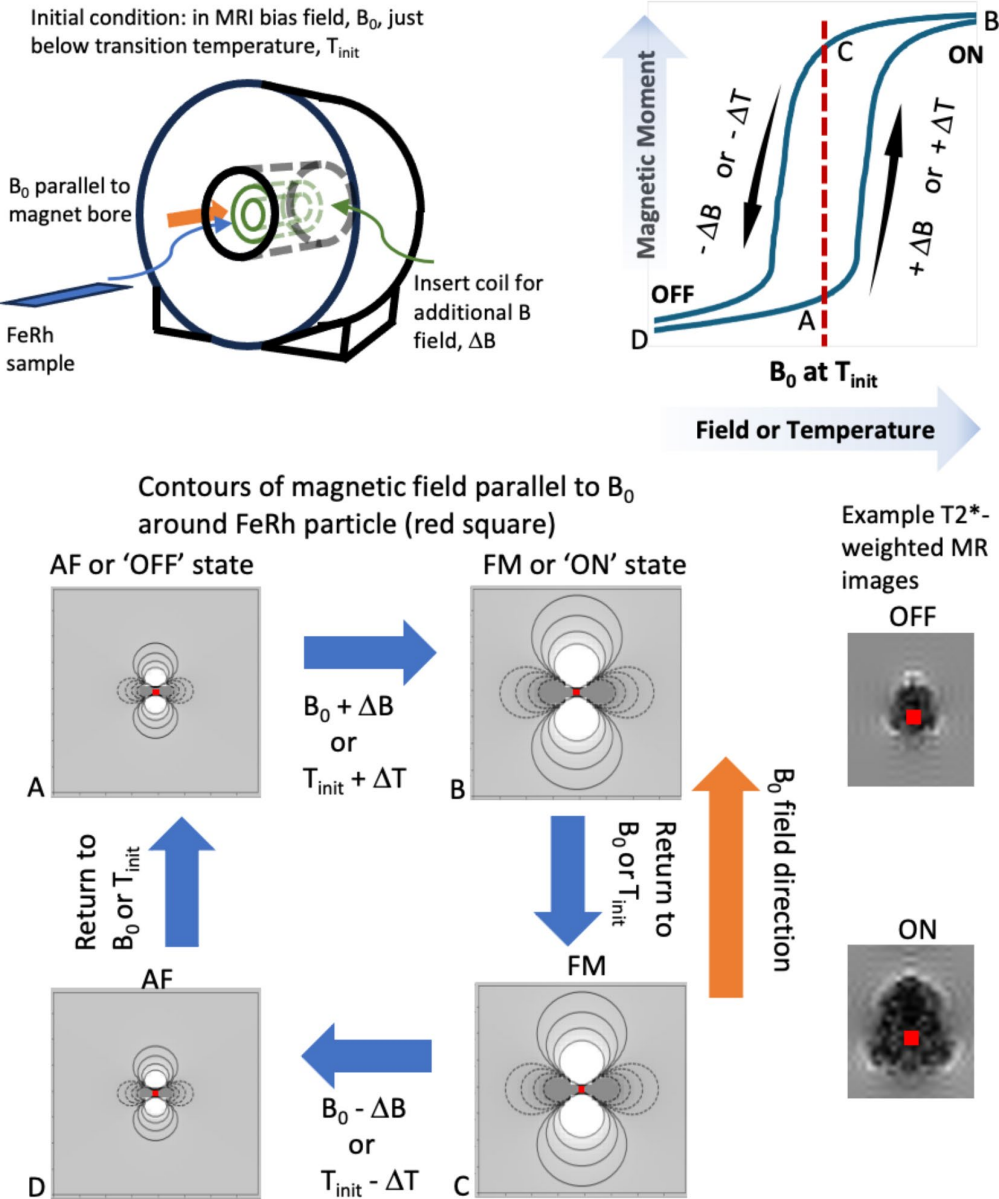
Schematic showing the contrast change for FeRh as it switches between AF and FM states. (**A**) The sample goes into the MRI scanner in the AF or OFF state below the transition temperature. (**B**) Either the temperature or the magnetic field may be increased to allow transition to the FM or ON state. (**C**). The temperature or magnetic field can be reset to their initial state, however the FM or ON state is retained due to the hysteresis. (**D**). Decrease of temperature or magnetic field returns the sample back to the AF or OFF state. MRI can take advantage of the OFF-ON transition with images sensitive to the local magnetic field disturbance (T2*-weighted), as shown.

Metamagnetic phase transitions present an interesting opportunity to develop switchable and tunable contrast agents suitable for MRI^1^. For example, Iron-Rhodium (FeRh) exhibits a large change in magnetic moment with a change in temperature and/or external magnetic field in field range that is readily available in MRI magnets and at physiological temperatures. For MRI, these materials show potential for experiments such as cell tracking^2,3^ where the large magnetic moment change would give rise to a dramatic shift in contrast when sensitized to local magnetic field variations (T2* contrast). In this case the strong variations in the magnetic field surrounding the particle leads to a loss of phase in the MRI signal of the surrounding medium and hence a darkening of the image, as shown in Fig. 1, with the characteristic pattern^4^. The MRI signal is very sensitive to this distortion and can be detected at a distance well beyond the size of the particle (greater than 50 times the diameter), often referred to as the ‘blooming effect’^5–7^. As an example, this allows a micron-sized magnetic particle to be imaged at MRI resolution of 100 μm^8^. Iron-oxide particles^9,10^ are commonly used as magnetic labels to generate the T2* contrast, however this contrast remains relatively constant. The ability to switch the label imaging contrast on and off with metamagnetic materials should allow for unambiguous detection. This would be useful for distinguishing them from the iron in blood or other iron-oxide particles, increasing the specificity of the label.

Iron-Rhodium (FeRh) exhibits a metamagnetic first-order phase transition, from antiferromagnetic (AF) to ferromagnetic (FM) when either external magnetic field or the temperature is increased^11^. There is an associated change in lattice volume, magnetoresistance, and entropy, which makes FeRh an interesting material for a number of applications, including magnetic refrigeration^12^. Of relevance to MRI is that the magnetic moment change is almost an order of magnitude over a small temperature rise or external magnetic field change. In a large bias field, such as in a high field MRI scanner, changes in the material composition can be used to tune applicable temperature ranges to physiological values^13^. As an estimate of the potential MRI contrast that metamagnetic materials offer, in the FM or high moment phase, defined here as ON for MRI contrast, FeRh has a saturation magnetic polarization of ~ 1.4T^14^, comparing favorably with iron-oxide (Fe_3_O_4_) at ~ 0.6T and pure iron which saturates at ~ 2T. In the AF or low moment phase of FeRh thin films, defined here as OFF for MRI, there is a residual magnetic moment amounting to ~ 10% of the FM phase^15^. Thus, there is a 10-fold change in magnetic moment from low moment OFF to high moment ON states. In the case of FeRh, there is also a large magnetic moment hysteresis at the phase transition, which enables the material to act as a switch for MRI with both the OFF and ON states imaged at the bias MRI field.

In prior experiments it was shown that bulk samples of FeRh can exhibit large contrast changes with a temperature sweep experiment at 4.7T MRI^1^, and then similarly with Lanthanum-Iron-Silicon^16^ which switches from high to low moment with increasing temperature, in reverse to FeRh, enabling the two to be distinguished^17^. In these prior publications, only large pieces were available with sizes down to ~ 100 μm in diameter. There is a question as to whether the metamagnetic materials will maintain their properties when scaled down to sizes suitable for cellular labelling. The magnetic phase transition has been demonstrated in FeRh for submicron stripes^18^, structures^19^ and even nanoislands^20^. Our current experiments leverage this work with an eye towards making cell size metamagnetic particles by patterning a thin film (~ 200 nm thick) into an array of various mesoscale sizes. MRI measurements were performed on this array to demonstrate that micron scale FeRh contrast agent can be produced and switched either with temperature or brief changes in magnetic field making it feasible to use metamagnetic materials for cellular and molecular MRI applications.

Previous studies generating MRI contrast with metamagnetic materials^1,17^ have only used temperature to switch the materials. However, FeRh switching can also be achieved by changing the bias field, which need only be temporary due to the inherent hysteresis. Here we present results showing that micron-scale Fe-Rh thin films can be switched with a temperature sweep or by using a shift in the MRI bias field, B_0_. For this purpose, a shielded B_0_ insert coil capable of ‘shifting’ the main MRI magnetic field by up to ± 0.77T for a few ms over a 25 mm-diameter spherical volume was used. B_0_ field shifters within an MRI that switched up to 0.2T have been used in contrast agent studies^21,22^. Here, shorter pulses are used to allow the higher magnetic field strength without generating significant heating in the resistive windings. The use of this pulsed B_0_ field relies on the fast switching of the FeRh magnetic moment with time. The hysteresis in the moment enables imaging to be done in both the OFF and ON states at the same MRI bias field, which was 4.7T in the present experiments. Figure 1 shows a schematic of how the experiment works.

## Methods

**Fig. 2:**
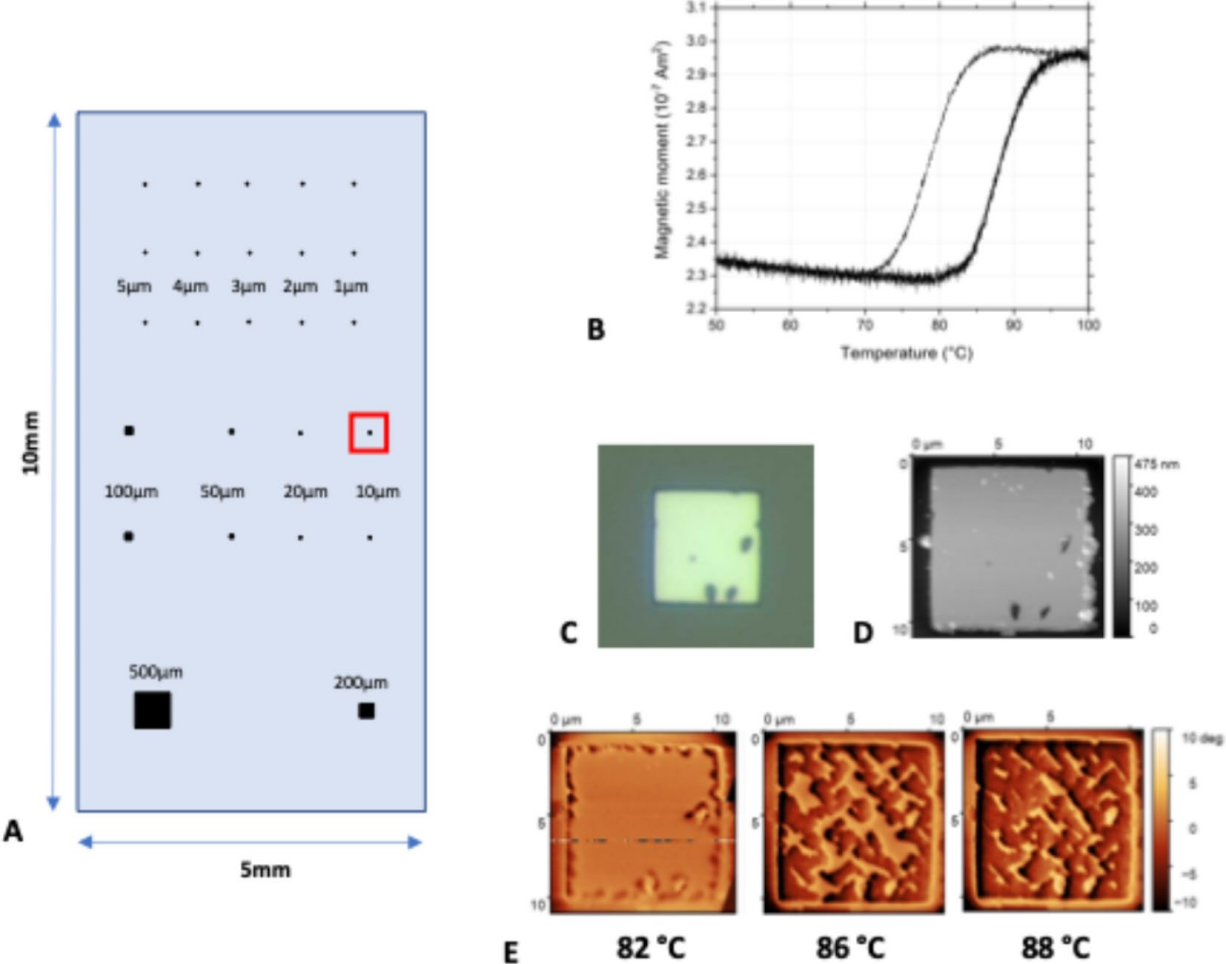
(**A**) Layout of the FeRh sample, starting at a 500-µm square down to 1-µm square, each with a thickness of 200 nm. (**B**) VSM measurements of magnetic moment during a temperature sweep at 1 T external field. The majority of the signal comes from the two largest squares. The high-moment state is considered ON for MRI, and low-moment state is considered OFF. (**C**) Optical image of a selected 10-µm square (red-circled in** A**), with a few minor defects. (**D**) Atomic force microscopy obtained topography image of the same square at 88 °C; (E) corresponding magnetic force microscopy images with increasing temperature. The change from AF (light areas) to FM (dark areas) phase takes place during heating. Distortion of the square shape due to thermal drift of the sample stage was corrected using the Gwyddion program^19,20^.

FeRh squares of 200 nm thickness were patterned onto a 5 × 10 mm^2^ MgO substrate with the layout as shown in Fig. 2A (see Supplementary 1 for full dimensions). An epitaxial 200-nm-thick FeRh film was first grown on MgO (001) single-crystal substrates at 450 °C and an argon pressure of 2 mTorr by d.c. magnetron sputtering using an equiatomic target. The film was post-annealed at 800 °C for 80 min and subsequently coated with a 2-nm Pt layer^23^. The FeRh film was patterned into microstructures by e-beam lithography and ion etching through a Ti/SiO_2_ mask. The sample was finally coated with a protective 100-nm-thick SiO_2_ film. The sample was characterized with vibrating sample magnetometer measurements at a 1T bias field, and with atomic and magnetic force microscopy (AFM/MFM). Atomic/magnetic force microscopy data were processed, and thermal drift was corrected in the open-source Gwyddion software^24,25^. The imaging was performed in conventional 2-pass lift mode with MESP v2 probes from Bruker.

The sample was immersed in water (lightly doped with 50 µM MnCl2) and imaged at room temperature at 4.7T and 11.7T MRI fields which was estimated to have the FeRh in the low moment, OFF, and the high moment, ON, states respectively. 3D gradient-echo MR images were acquired with an echo time (TE) of 10 ms to provide T2* weighting. The imaging slab was aligned manually to the surface of the FeRh array. The image resolutions were 50 and 100 μm isotropic, at 11.7T (Bruker AV-NEO console) and 4.7T (Bruker AV-III console) respectively. Other imaging parameters were Repetition Time (TR) = 30 ms, Flip Angle (FA) = 15°, Matrix size = 512 × 192 × 128 at 11.7T and 256 × 96 × 64 at 4.7T, Acquisition Bandwidth (BW) = 50 kHz at 11.7T, and 25 kHz at 4.7T. All analysis was performed on the image slice just above the sample array. The acquired images were compared with simulations, generated using the method of Bakker et al.^26^, assuming the polarization in the ON state is 1.4T and the OFF state being 10% of this at 0.14T. The square structures of the array were approximated by spheres of equivalent volume. The simulations model the effect of magnetic susceptibility differences between the magnetic particle and an imaging medium, such as water, and then computes the relative contrast on a T2*-weighted image.

To observe the temperature dependent magnetic phase transition of the FeRh particles, we performed a temperature sweep at 4.7T. The sample tube temperature was controlled with a circulating water bath, and serial images were acquired during heating and cooling cycles over a range from ~ 20–60 °C. For this experiment we imaged using a 3D flow-compensated gradient-echo sequence with 150 μm isotropic resolution, resulting in a time resolution for the temperature sweep of ~ 4 min (TR/TE = 25/10 ms, Flip Angle = 10°, matrix size = 192 × 128 × 70, zero-filled to 384 × 256 × 70, BW = 25 kHz). A home-built quadrature birdcage coil, with id of 26 mm was used for acquisition at 4.7T (supplementary 2). Temperature readings used in this experiment were taken from a fiber-optic temperature probe (OTP-M, Opsens, Quebec, QC, Canada) that was attached to the platform holding the sample. To save time, the sample was continuously heated or cooled and the temperature reading was taken at a time corresponding to the middle of the scan when the center of k-space was acquired.

After determining the transition temperature from the temperature sweep, we performed a field switching experiment using an actively shielded B_0_ insert coil (Resonance Research Inc, Boston, MA). The insert had a 40 mm ID and 150 mm OD and was designed to generate ± 1T pulses with a current of 355 A over a 25 mm-diameter spherical volume. The insert was driven with a repurposed gradient amplifier (Copley 265P), with an Agilent 33,250 A used to input the waveform. With this arrangement, the insert could be driven with up to 275 A, generating ~ 0.77T. The insert dimensions are suitable for mouse imaging and to fit inside one of the standard imaging gradient sizes used for preclinical imaging. The nominal inductance was 4.94 mH with a resistance of 886 mΩ. Performance of the insert was verified by applying low amplitude direct current and measuring NMR spectra from a small water sample (supplementary 3). This method was also used to determine the field polarity. Temperature of the B_0_ insert was monitored with PT100 sensors built into the coil windings and connected to a raspberry Pi microcontroller equipped with an RTD hat (Sequent Microsystems, Cupertino, CA). A temperature rise of less than 0.05 °C was observed for all experiments (supplementary 3).

The FeRh particle array sample was warmed to just below the metamagnetic transition temperature point determined from the temperature sweep. Temperature stability was monitored for 15 min. An OFF-ON MRI experiment was performed by acquiring MRI in the low moment off state and a 275 A Gaussian pulse, generating a maximum of 0.77T, was applied for 25 ms to switch the particle to the high moment, ON state. Due to hysteresis the ON state remains even though the field is returned to 4.7T for image acquisition. A pulse of opposite polarity (-0.77T) was applied to overcome the ON hysteresis to create the low moment OFF state which was maintained when returning to 4.7T enabling an OFF MRI to be obtained. This OFF-ON-OFF cycle was repeated three times to confirm reproducibility and demonstrate controllable particle image contrast switching.

The images from each set of experiments were registered using the edges of the sample substrate as a guide. This was mainly to account for frequency shifts from the temperature sweep, as well as some settling of the sample when the circulating tubing used to maintain temperature got warm. The image slice closest to the FeRh array after registration was used for analysis. All image analysis was done in Python and MIPAV (https://mipav.cit.nih.gov).

## Results

Vibrating sample magnetometer measurements (VSM) in a field of 1 T during a temperature sweep are shown in Fig. 2B. An optical image of one of the 10-µm squares (red-square in Fig. 2A) of the final sample is shown in Fig. 2C. In Fig. 2D and E, respectively, AFM and MFM images are shown for the corresponding square, where the transition from AF to FM can be observed with the shading change in the images as temperature is increased. Based on this data it was estimated that the FeRh thin films would switch at 4.7T at a temperature below 60 °C or with a B_0_ field increase of about 1T near but below the transition temperature.

**Fig. 3:**
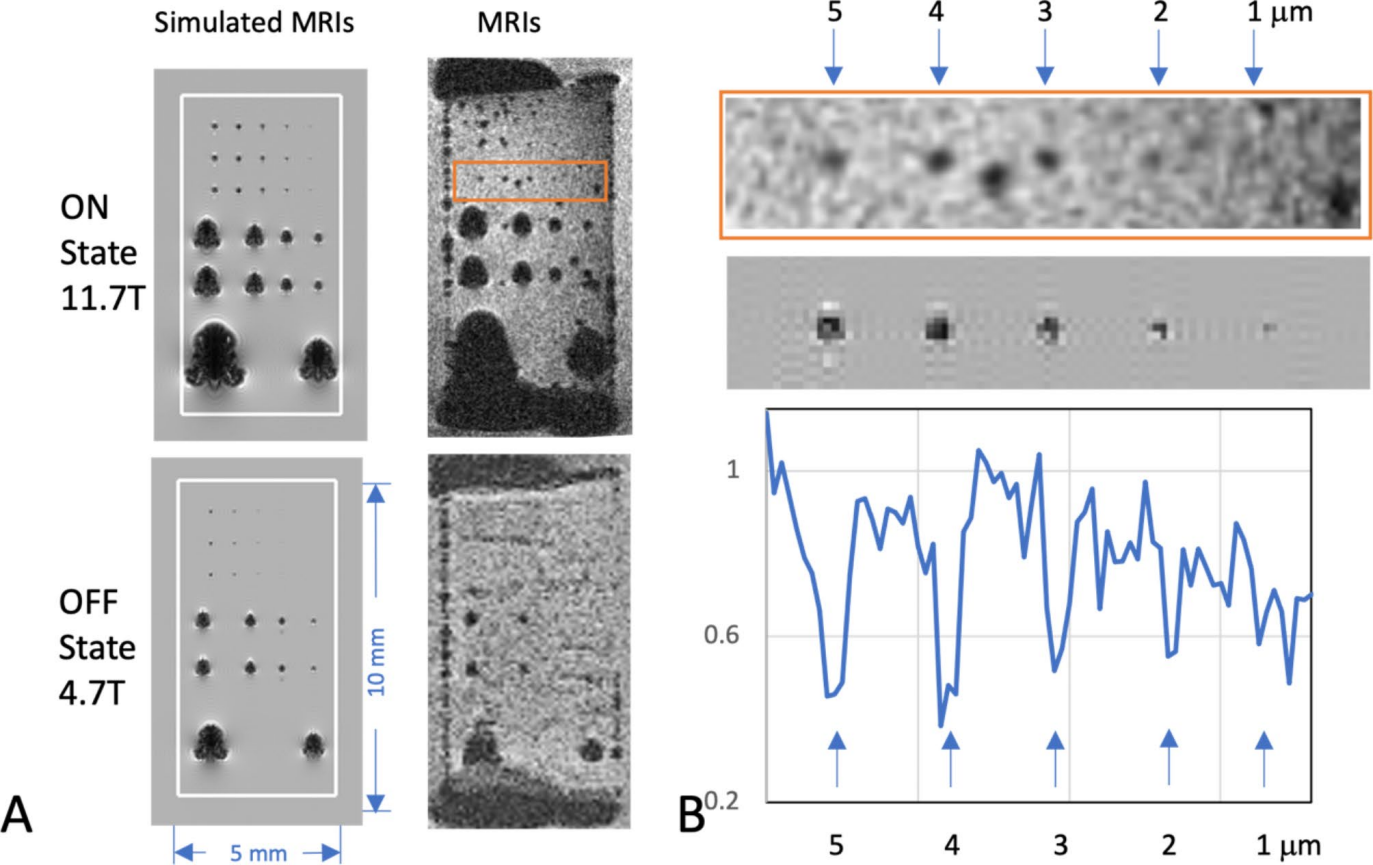
(**A**) Simulated gradient-echo MR images (TE = 10 ms) in the ON and OFF state. The white line represents the outline of the array substrate. These are compared with images from 11.7T at 50 μm isotropic MR image from the plane closest to the sample, and with images at 4.7T at 100 μm resolution (both images acquired at room temperature). Despite the artifacts from the ends, the layout can be seen clearly down to structures at 2–3 μm at 11.7T. This is best shown in the zoomed-in region in (**B**), along with a plot of the intensity profile through the center of the particles. The marked change is consistent with sample being in the ON state at 11.7T and the OFF state at 4.7T, correlating well with the simulated images. All images were 3D gradient-echo with TE = 10 ms. The direction of the MRI field is up-down in all images.

In Fig. 3A, simulated 50 μm gradient-echo MR images (TE = 10 ms) are shown in both the ON and OFF state assuming a 10-fold change in moment. The simulations compared well with gradient-echo MR images of the thin film patterned FeRh sample at the OFF state in the 4.7T MRI (100 μm isotropic) at room temperature (~ 20 °C) and for the ON state obtained in an 11.7T MRI at 50 micron resolution at room temperature. The simulations correlate with the sample being in the ON state at 11.7T and in the OFF state at 4.7T, which was expected from the characterization of the material. Simulations with the array overlaid are shown in Supplementary 4.

From the initial baseline MRIs in Fig. 3B particle MRI signatures from the ON state could be detected. There are artifacts likely from air bubbles being trapped under the sample slide and the susceptibility differences between the sample substrate and surrounding water. Despite this, the FeRh thin film pattern is readily visible, down to the 10 μm particles in the 100 μm resolution images at 4.7T and down to 2–3 microns in the 50 μm image at 11.7T. This is shown in the inset where a row from the small group is compared with the simulations, with the intensity profile through the center of the row for the MRI. At 4.7T, the sample signatures are smaller (approximately half the size in linear distance), which is typical of the OFF state. Image resolution was 100 μm, therefore only 50 μm or larger FeRh thin film squares were visible in the OFF state at 4.7T.

**Fig. 4:**
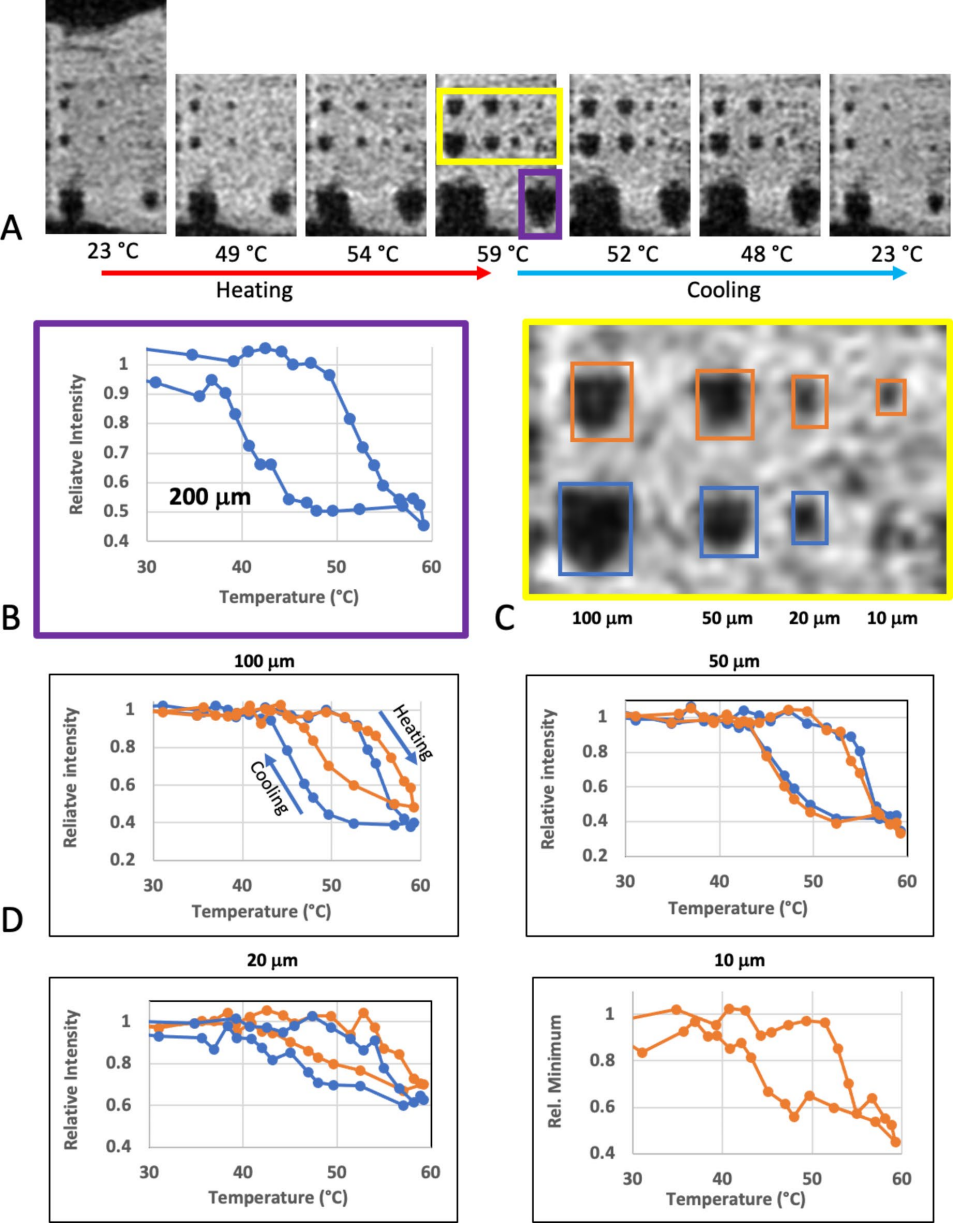
(**A**) Gradient-echo MR images from a temperature sweep at 4.7T. The signatures of the structures from 500 μm down to 10 μm clearly enlarge after heating to > 50 °C, and then return to baseline during cooling with a lag from the hysteresis as expected. Image resolution here was 150 μm isotropic with 10 ms TE, to get a time resolution of 4 min. (**B**) Normalized MRI mean signal intensity plot of an ROI around the 200 μm structure. (**C**) ROIs from the 10–100 μm structures used for the normalized mean intensity plots in (**D**) The hysteresis curves are as expected for FeRh, although there are some differences in the transition temperature. One of the 10 μm structures is obscured by debris and is not plotted.

The temperature sweep at 4.7T, starting around room temperature (which was determined to be in the OFF state from the baseline images), up to 59 °C demonstrated a significant change in the particle MRI. Selected images from this temperature sweep are shown in Fig. 4A (video, Supplementary 5). The mean signal intensity of an ROI drawn around the 200 μm particle was measured. This is plotted in Fig. 4B. We note the characteristic curve of FeRh switching to a high moment ON state as was observed in previous studies^1^. For the FeRh thin films the transition at 4.7T MRI started at a temperature of approximately 48 °C. Similar plots for the smaller particles (100 μm or less) are shown in Fig. 4D, with plots of mean signal intensity of small ROIs drawn around each particle (indicated in Fig. 4C). It is noted that the transition temperature varies slightly across the array, most readily observed in the 100 μm pair from Fig. 4D.

**Fig. 5:**
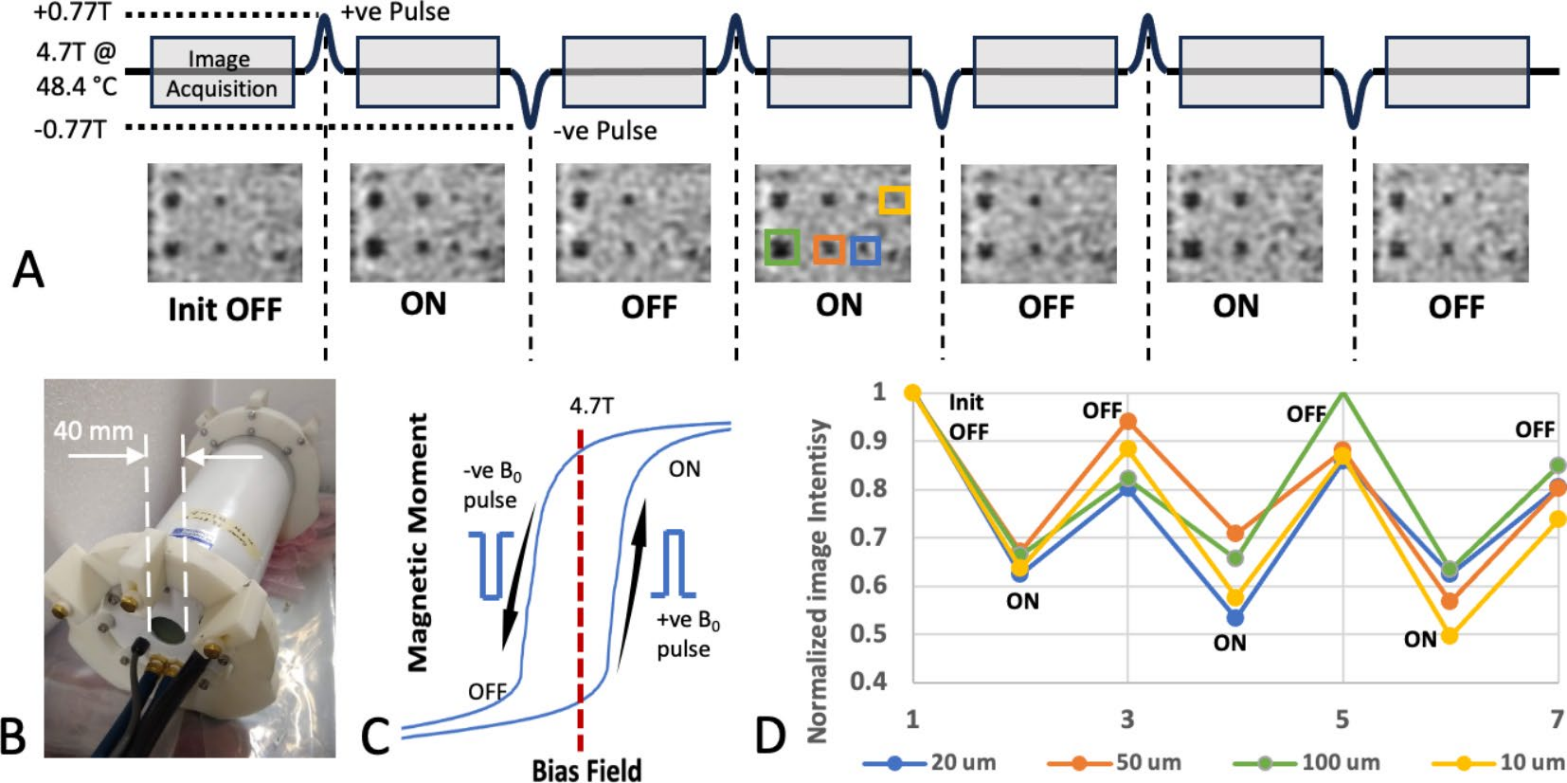
Particle switching demonstration with the intermediate structures. With the temperature held at 48.4°C, the image series in A was acquired. Following an initial image a 25 ms Gaussian pulse of 0.77T was applied with the B_0_ insert (shown in **B**), effectively raising the magnetic field to 5.47 T very briefly over the sample. Following a subsequent image, a -0.77T amplitude pulse was applied. The hysteresis curve, shown in C, switches the magnetic moment between ON and OFF states, allowing a subsequent MRI acquisition with switching T2* contrast. This was repeated three times to obtain the image series shown in A. Plots of the mean image intensity for four of the particles are shown in (**D**), using the ROIs shown. For the 10 μm particle, the minimum intensity in the ROI was used.

With the temperature held at 48.4 °C, an in situ field shifting ON-OFF particle contrast switching experiment for some of the smaller structures (10–100 μm) was performed, shown in Fig. 5A (video, supplementary 6). These images were acquired after pulsing the B_0_ insert shown in Fig. 5B while the particles remain in the ON or OFF state due to magnetic hysteresis (Fig. 5C). Plots of the image intensity of four selected squares are shown in Fig. 5D. The blinking of the particles through an OFF-ON-OFF-ON cycle enables distinguishing the particles from the spurious T2* artifacts caused by air bubbles and manufacturing defects.

## Discussion

The switching T2* contrast associated with the metamagnetic phase change in magnetic moment in a thin film FeRh sample of varying sizes was demonstrated both with in situ temperature changes and in situ magnetic field changes. This clearly demonstrates that the eventual goal of making a switchable MRI contrast agent as a cellular label is possible with FeRh. Indeed, a 10 μm square with 200 nm thickness element has the same volume as a 3.2 μm diameter sphere, a volume already demonstrated to be suitable for cellular labeling^8^.

The simulations corresponded well with the ON and OFF state measurements from the 11.7T and 4.7T magnets respectively. The array elements were approximated as spheres of equivalent volume, which will lead to decreased accuracy as the linear dimension increases. For example, the 500 μm square particles covers 10 × 10 image pixels, which may be significant. Saturation magnetic polarization values were taken from literature^14^ and it would be more accurate to use measured values from the film. It should be noted that effects of diffusion are not considered which may limit accuracy at higher imaging resolutions.

The temperature response curve (Fig. 3B and C) of the FeRh array used in the present studies and the bulk materials used in previous experiments is highly dependent on the size of the structures and strain imposed by the substrate^19^ making it difficult to predict at what MRI fields and temperatures are best to operate. This variability should be possible to control with microfabrication techniques being used to make a single size. Indeed, this variability could enable different particle sizes and materials to create different labels, for example by requiring different B_0_ field shifts. Ultimately, this can be used to label different cell-types.

Although a labor-intensive technique, microfabrication will be able to generate significant quantities of particles. One previous study^35^ generated 2 μm iron discs spaced 10 μm apart in a grid which could be released by dissolving a sacrificial layer. In this case, one wafer yielded > 100 million particles, sufficient for numerous experiments in our hands. Thin-film patterning could also be complemented with a solid-state dewetting route as well, which is faster, more economical, and scalable. High-quality FeRh structures can be prepared by solid state-dewetting of epitaxial films, which could be released from the substrate into a solution^20^.

Experiments using a B_0_ insert to shift the magnetic field ± 0.77T within the 4.7T MRI were successful. Using a magnetic field pulse is a preferred way to switch the metamagnetic materials for biological experiments for a couple of reasons. First, it is generally not feasible to heat a biological sample by 10 °C or more to cause the moment switch, although there are some experiments where it might be useful such as localized heating^27^. Secondly, susceptibility artifacts from other sources can change with temperature, adding an unnecessary confound to image interpretation. For example, we observed susceptibility artifacts from gas bubbles at the higher temperatures used in our experiments.

The current field shifter is only able to hold small samples, e.g. up to the size of mouse, and to build a B_0_ shifter capable of ± 1T is technically challenging. There are at least two possible solutions for this. One approach would be to use a device like a transcranial magnetic stimulation (TMS) coil to generate large localized B_0_ changes. Versions of TMS coils have been implemented in MRI scanners^28^. These can generate Tesla-scale magnetic fields at their surface^29^, although the field falls off quickly limiting use to near the coil. A second approach would be to use a main magnet which can change magnetic field relatively quickly by more than 1 Tesla. There are new commercial preclinical magnet options which can change magnetic field on the Tesla scale in about 20 min (Maxwell magnet, Bruker Biospin and Drymag, MR Solutions). Although slow, this type of magnet would be useful for a proof-of-concept study in larger animals. There is also a long history of shifting magnetic fields very quickly for performing pre-polarized MRI^30^. These tend to be at lower field and appropriate materials would need to be used to control the metamagnetic state at these lower fields.

We were able to perform a simple ON-OFF type experiment with the B_0_-shifter to detect particles down to the 10 μm squares. A 10 μm particle effectively blinks on and off (best seen in video Supplementary Fig. 5), and this is how we envision an eventual cell tracking experiment to work, with multiple on-off cycles and with the size and moment change of the metamagnetic particles tuned to the intended image resolution. From simulations, we expect that a FeRh particle centered in a voxel, sized to give a 50% drop in signal (relative to background) in the ON state will have about a 5% drop in signal due to the low moment in the OFF state making it difficult to detect.

There were limitations in the way we used the B_0_ shifter for these experiments. The shifter is a an efficient coil (2.8 mT/A) and therefore noise from the shifter disrupts the MRI when connected to the amplifier. For the presented tests we took an image, opened the magnet bay door, plugged in, and pulsed the shifter, and then unplugged it and closed the magnet bay. Filtering the inputs or installing a physical quick disconnect would enable use of the shifter without opening and closing the RF shield but it is unclear if any filters will be good enough to suppress noise from the amplifiers. Therefore, a remotely operated disconnect will likely be a better solution. The 0.77T shift only covers about 75% of the moment range available for switching. The B_0_ insert is rated to generate a 1T field but we were limited by available power from the amplifiers used for these initial experiments. The fact that the temperature did not rise significantly in the shifter for the implemented pulse lengths and duty cycle used makes it possible to deliver more power to get the full change in moment.

It is likely that the 25 ms Gaussian pulse duration (> 95% amplitude for 2 ms) used with the B_0_ shifter was sufficient to fully switch the moment of the particles, with the pulse being slow enough that we are operating in the quasistatic regime. However, no tests were performed to ensure that the duration of the pulse was sufficient to complete the switching of the material. This can be done by adding a plateau at the maximum amplitude of the pulse for slightly longer durations to ensure the image contrast does not change. The results were consistent with the 8 °C /T phase transition temperature shift^31^ with FeRh (Supplementary 7), which is evidence that the pulse length used was sufficient to switch the array.

Although outside the scope of this work, biocompatibility of the material will have to be considered for future long term biological experiments. Iron is an essential element, although it can be toxic in large amounts^32^. Iron based contrast has been used in many pre-clinical MRI studies and the FDA has approved two iron oxide formulations for clinical use. Rhodium has no biological function, and is generally considered of low toxicity, although is toxic at large concentrations^33^. Previous studies have indicated a low cytotoxicity for FeRh particles^34^. It is likely that that any FeRh particles used for biological studies will be coated with a biocompatible surface for example with a metal, such as titanium or gold, during microfabrication^35^. Gold allows for functionalizing the particles through thiol chemistry^36^. An alternative would be to coat the particles after microfabrication with biocompatible materials such as dextran as is presently done with iron-oxide particles.

FeRh allows for interesting biological applications that are compatible with MRI detection. For example, it is possible to take advantage of the inherent magnetocaloric effect of FeRh whereby the material undergoes a change in temperature during the magnetic phase transition in response to a bias field change. This effect has been used to test drug release from FeRh attached to a thermally sensitive polymer^37^. The presented B_0_ insert will be useful in triggering the magnetocaloric effect for these types of applications.

The results presented at 4.7T were proof of principle. However, 4.7T fields are not useful for biological experiments of this sample due to the high temperatures required to enable the B_0_ shifter to switch the particles from OFF to ON. The transition temperature for FeRh films decreases by approximately 8 degrees per Tesla increase in bias field^31^. Therefore, an increase in the field from 4.7 to 7T should decrease the switching temperature by ~ 18 °C, from 48 °C used here at 4.7T, to ~ 30 °C at 7T. 7T MRI is widely available for both preclinical and clinical studies. Although this temperature is now lower than body temperature, rodents and cells can readily survive these temperatures. These higher fields will also give a higher SNR due to the higher field strength and lower sample temperature. For use at 37 °C, either the specific formulation of the particles will have to be altered, or the bias magnetic field used for the experiments will have to be approximately 6T, which is a field not commonly available for MRI but readily produced.

In summary, the present work has demonstrated that micron scale patterned thin films of FeRh can be used for switchable MRI contrast. Both temperature and B_0_ shifts were able to repeatedly and reproducibly switch the FeRh microstructures from a low moment MRI OFF state to a high moment MRI ON state. The B_0_ shifter allowed taking the contrast through OFF-ON-OFF cycles adding specificity to the determination of the presence of the FeRh MRI contrast and demonstrating switching potential for metamagnetic particles at a size suitable for cell labeling in MRI.

## Electronic supplementary material

Below is the link to the electronic supplementary material.

Supplementary Material 1

Supplementary Material 2

Supplementary Material 3

## Data Availability

The datasets used and/or analysed during the current study available from the corresponding author on reasonable request.
