# Supplementary material for: Field switching of microfabricated metamagnetic FeRh MRI contrast agents
Source: Sci Rep. 2025 Jan 22;15:2779. doi: 10.1038/s41598-025-85384-6 (PMC11754896; doi:10.1038/s41598-025-85384-6)
Supplement: Supplementary file 3 — Supplementary Material 3 [file 41598_2025_85384_MOESM3_ESM.pdf]

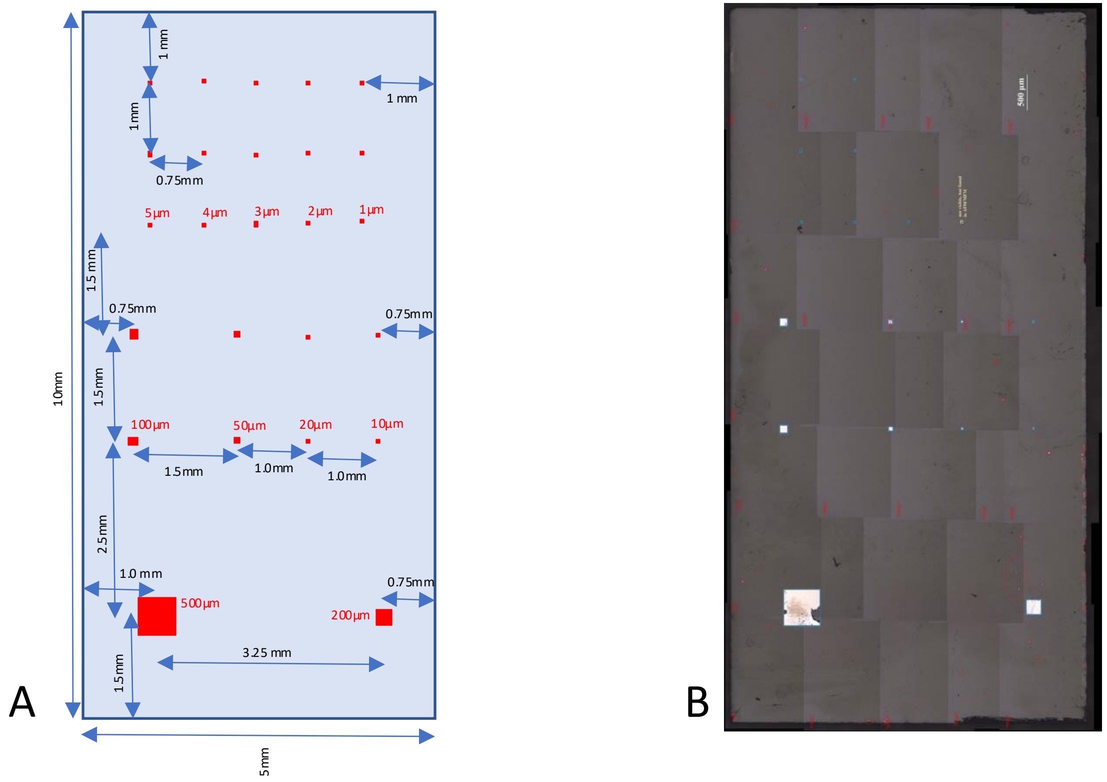

Supplementary 1

A. Full dimensions of the FeRh array. Structures below 100 µm are represented by the same square for clarity. B. Optical image of the array.

Supplementary 2 – Experimental setup

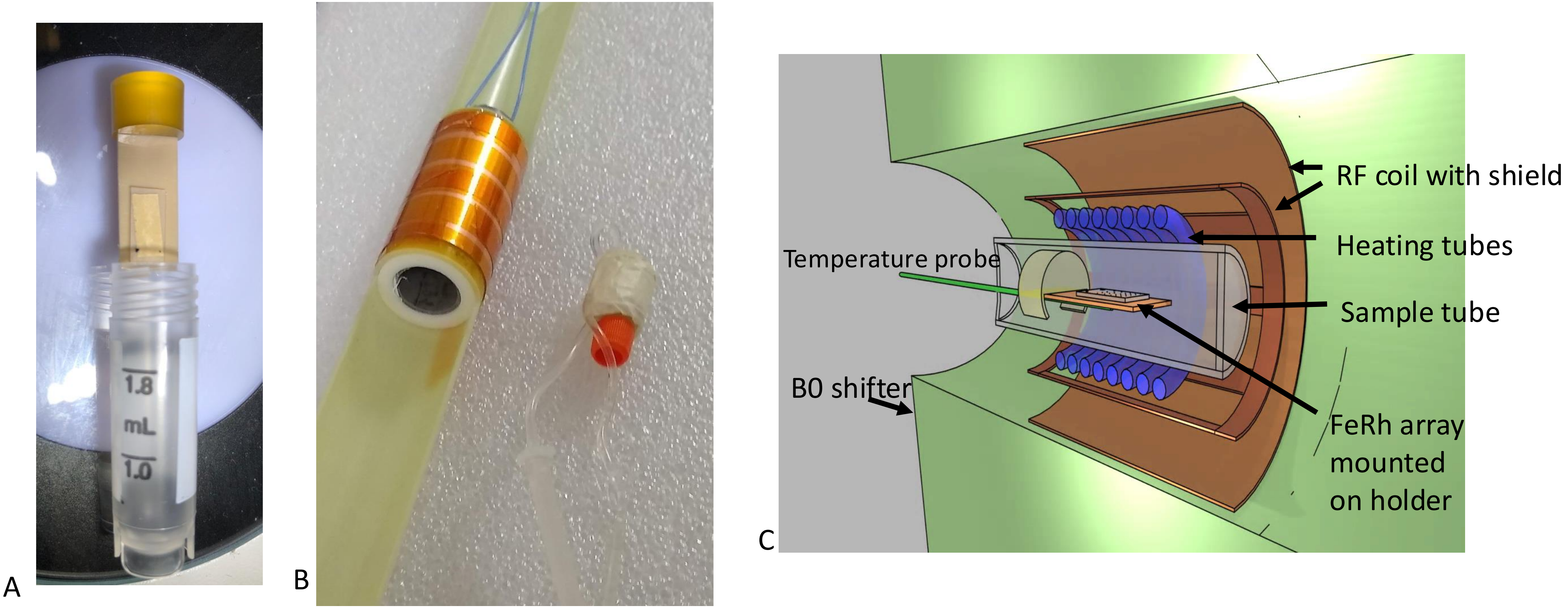

A. Picture of sample mounted on holder. B. Rf coil and sample with surrounding tubing for temperature control. C. Cutaway schematic rendering of the experiment setup.

B0 Shifter calibration – supplementary figure 3

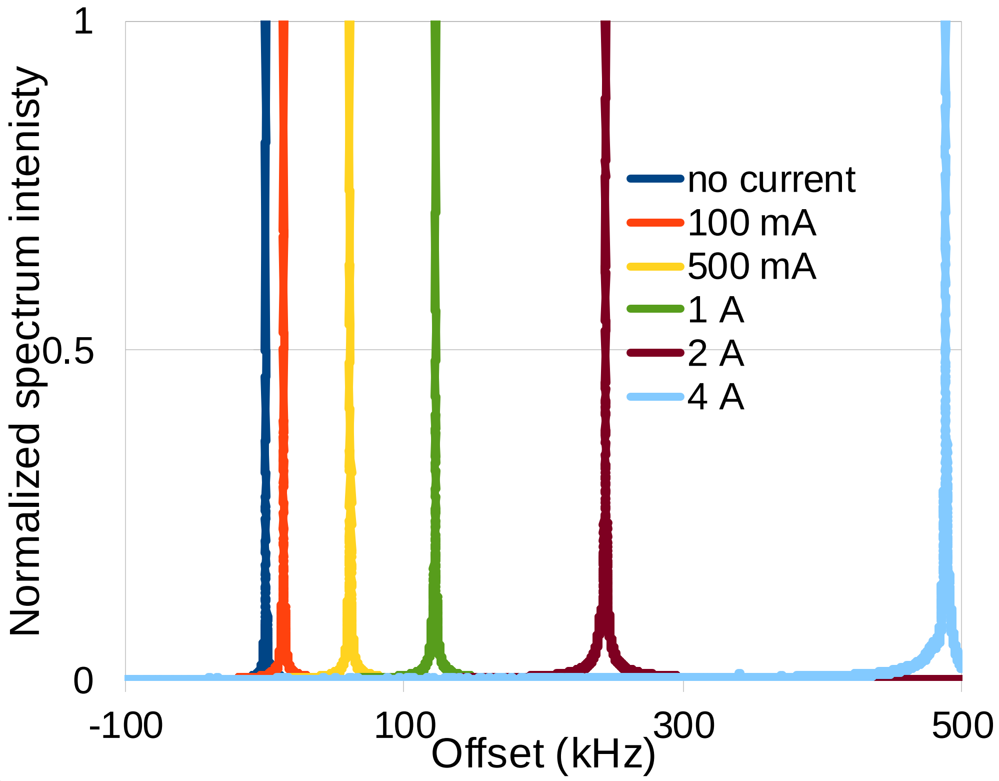

A

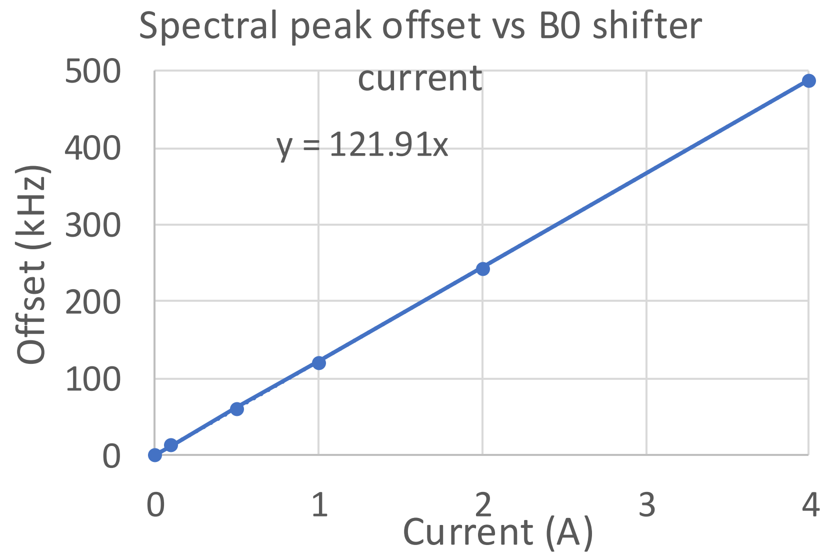

B

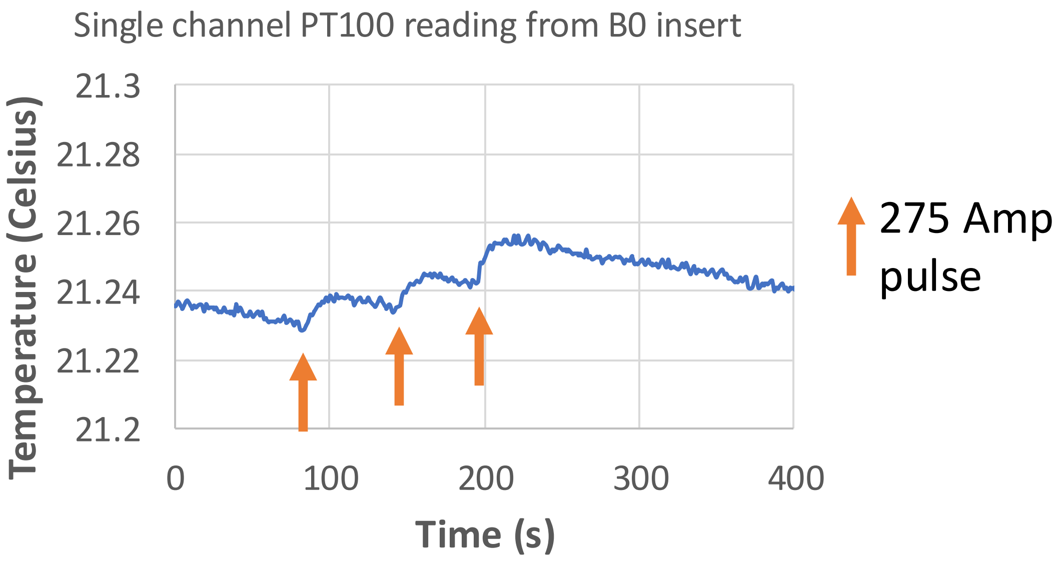

C

Calibration of the B_0 insert. A. NMR spectra of water sample with increasing amounts of current in the B_0 coil. B. Plotting the peak offset against the current give ~1T with 355A, per the manufacturer specifications. C. Temperature rise for one PT100 during 275A pulses.

Supplementary Figure 4

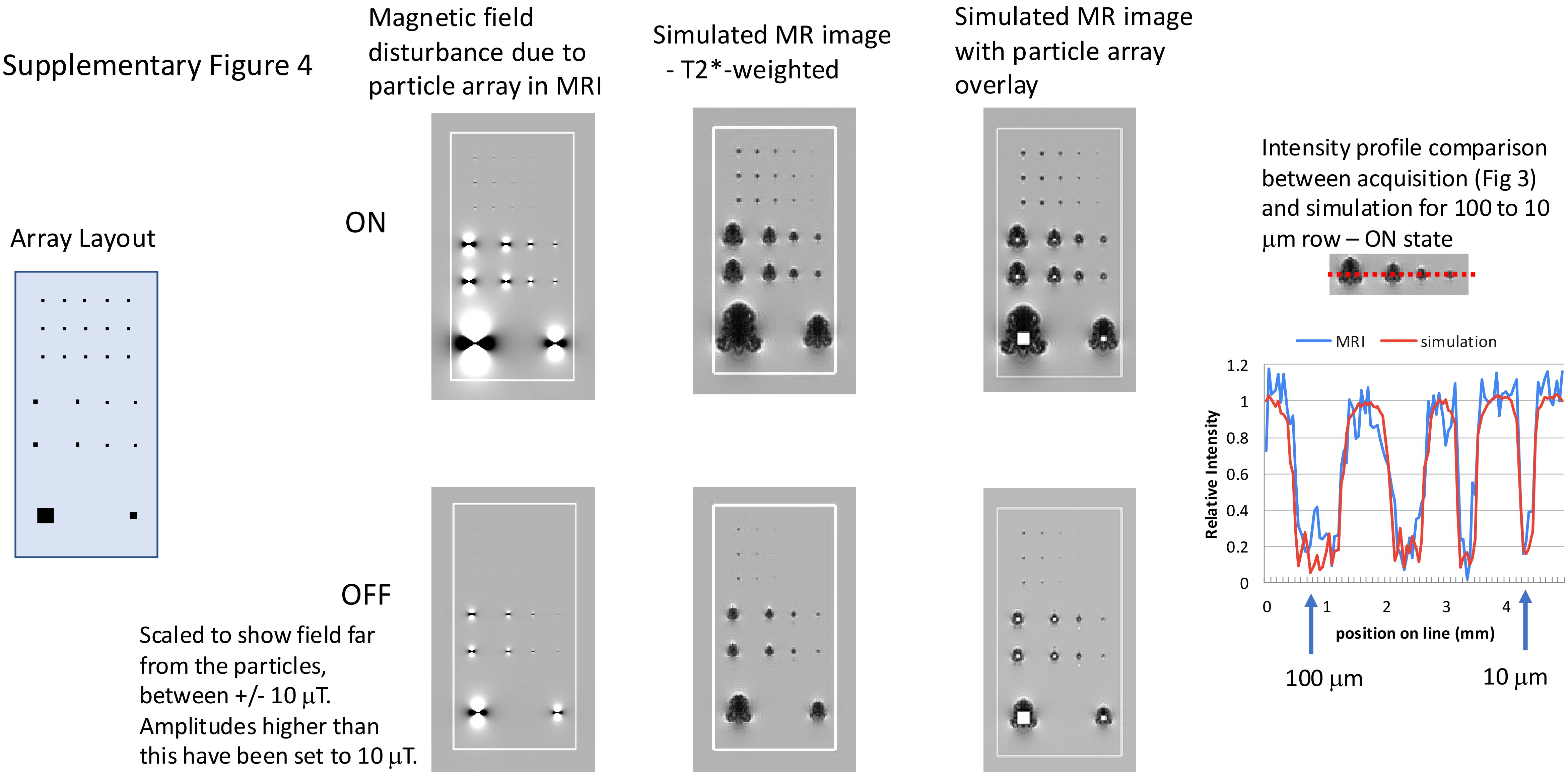

Simulations of the magnetic field dispersion around a FeRh array in ON and OFF states. This can then be used to simulate the expected MR image. An overlay of the particle array on the MR image is shown in the final column to show the position of the particles relative to the image. A profile comparison with shows excellent agreement with the acquired MRI.

Supplementary Figure 5 – temperature sweep

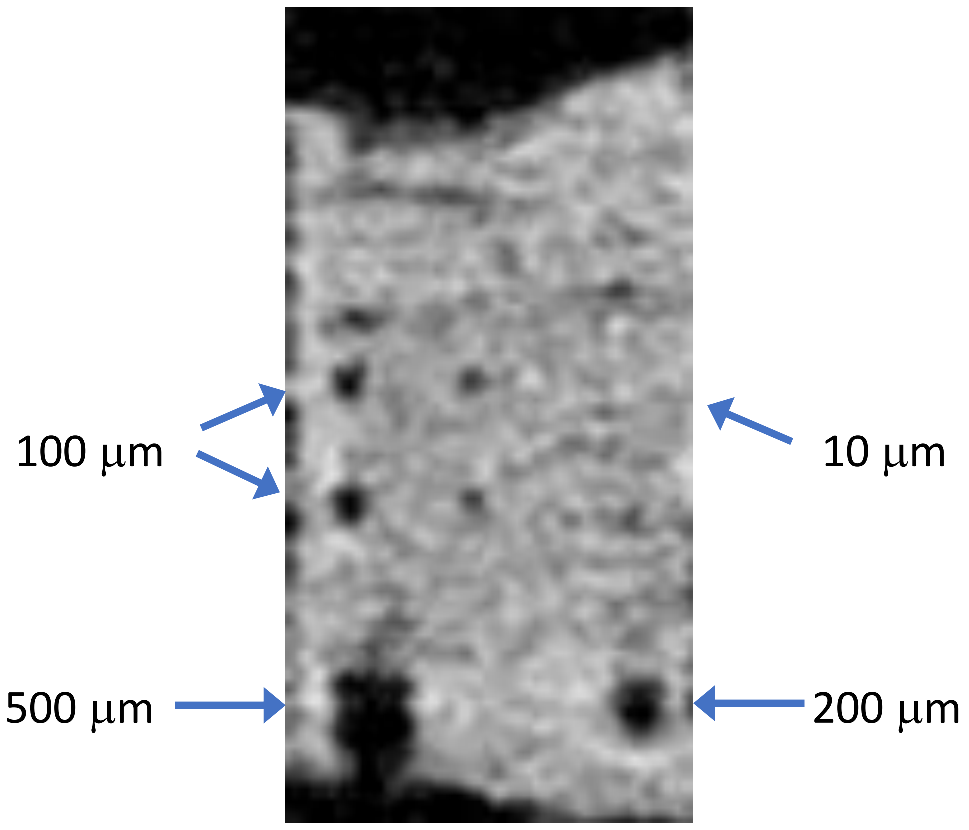

Supplementary Figure 6– B_0 shifter ON-OFF sequence

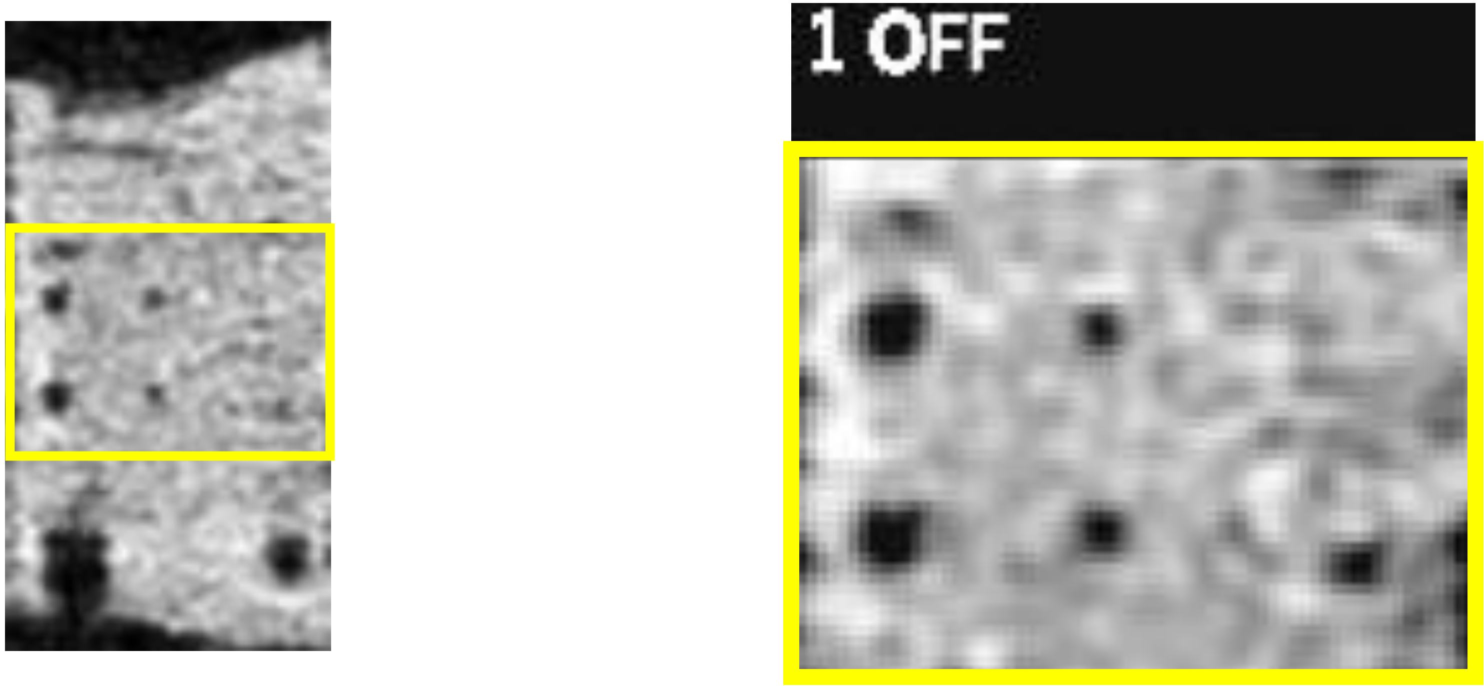

Supplementary Figure 7 – comparing temperature increase with positive field shift

All images acquired at 4.7T

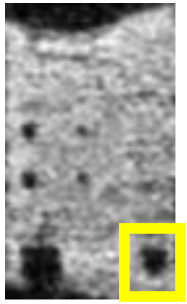

Confirmation of 8K/T for 200 um structure
0.74T = 5.9K

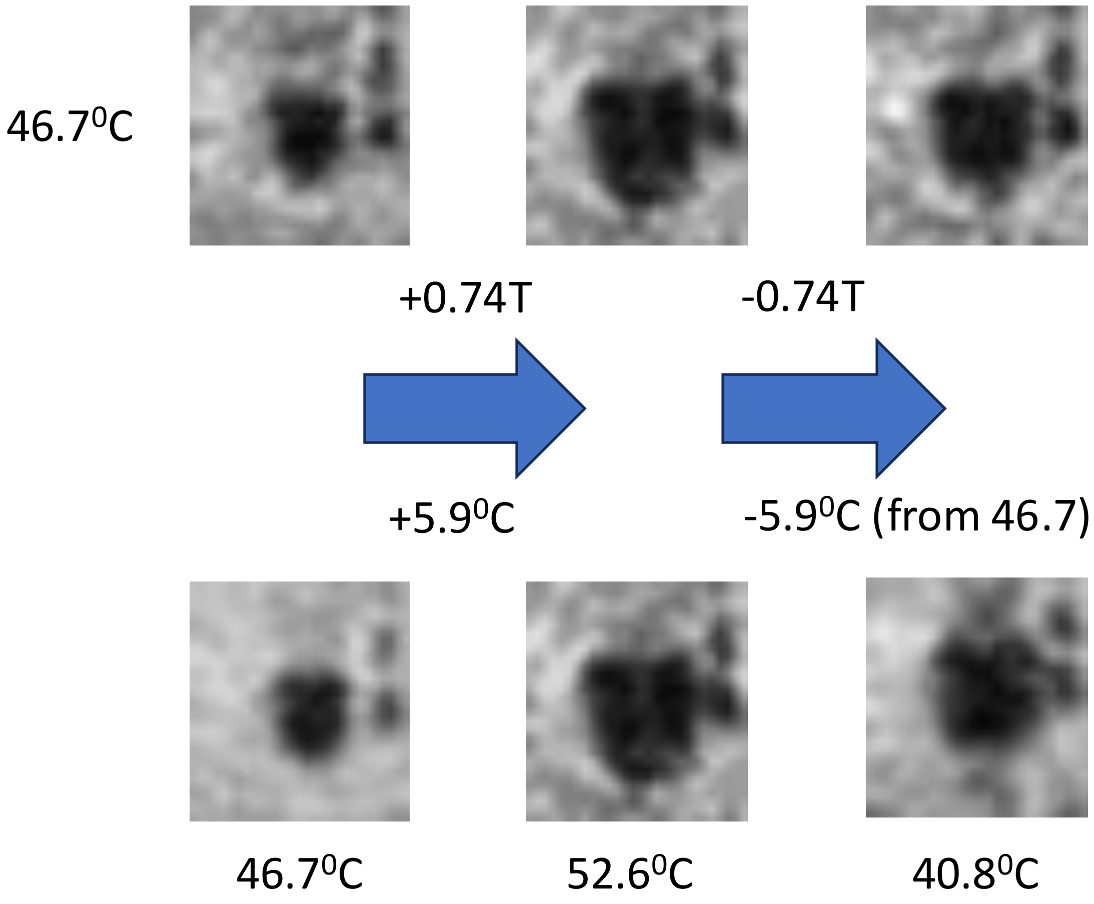

Images from linear interpolation of temperature sweep data

Comparison of images linearly interpolated from temperature sweep with images before and after B0 pulse. Qualitatively the images compare well, confirming the 8K/T shift for our sample, and also indicates that our pulse length is sufficient to fully switch the FeRh.
